# Interprofessional Team Training With Virtual Reality: Acceptance, Learning Outcome, and Feasibility Evaluation Study

**DOI:** 10.2196/57117

**Published:** 2024-11-04

**Authors:** Andrea N Neher, Rafael Wespi, Benjamin D Rapphold, Thomas C Sauter, Juliane E Kämmer, Tanja Birrenbach

**Affiliations:** 1 Department of Emergency Medicine, Inselspital, Bern University Hospital, University of Bern, Bern, Switzerland; 2 Graduate School for Health Sciences, University of Bern, Bern, Switzerland; 3 Department of Health Professions, Division of Nursing, Bern University of Applied Sciences, Bern, Switzerland

**Keywords:** medical education, simulation, virtual reality, VR, emergency medicine, interprofessional team training, nursing students, medical students, evaluation study, assessment, effectiveness, patient care, simulation-based training, hemorrhage, epileptic seizure, headache

## Abstract

**Background:**

Effective interprofessional teamwork is vital for ensuring high-quality patient care, especially in emergency medicine. However, interprofessional education often fails to facilitate meaningful interaction among health care disciplines. It is therefore imperative to afford early opportunities for cultivating interprofessional teamwork skills. While in-person simulation-based training has been shown to improve performance, this is resource-intensive, especially if it involves multiple professions. Virtual reality (VR)–based training is an innovative instructional approach that demands fewer resources and offers the flexibility of location-independent learning.

**Objective:**

This study aimed to develop and evaluate the acceptance, learning outcome, and feasibility of an interprofessional team (INTEAM) training course that included a VR simulation of a neurological emergency case.

**Methods:**

This 1-group study used a pre- and posttest design to evaluate the 2-hour INTEAM training course for nursing and medical students. The course included an e-learning part, VR simulation, and debriefing. The main learning objectives were derived from the entrustable professional activity 6, namely to handle a common problem in emergency medicine (headache due to subarachnoid hemorrhage and epileptic seizure) that requires interprofessional collaboration, including a structured handover. We used validated and self-constructed questionnaires, pre- and posttests, and open questions to assess the acceptance, learning outcome, and feasibility of the course.

**Results:**

The data of 42 students (21 nursing and 21 medical students) were analyzed and showed good usability in the System Usability Scale (median 72.5, IQR 65‐80). The perception of usefulness (median 6, IQR 5.8‐6.9) and ease of use (median 5.9, IQR 5.1‐6.3) was good among all students. There was a significant increase in the handover performance from pre- (median 8, IQR 6‐9) to posttraining (median 8, IQR 7‐9; *z*=−2.01; *P*=.045; *r*=0.33) and of the confidence in caring for patients with seizures (median 3, IQR 2‐3 and median 3.5, IQR 3‐4, respectively; *z*=−3.8; *P*<.001; *r*=0.60). In 67% (14/21) of the simulations, technical issues occurred, but all simulations could be carried out completely.

**Conclusions:**

The new INTEAM training course was well received by nursing and medical students. The handover skills and confidence in caring for patients with seizures were improved after the course. Despite technical challenges with the VR simulations, none required termination, and this demonstrates that our approach is feasible. These promising results encourage the use of VR simulations for team training in the education of nursing and medical students.

## Introduction

In all branches of medicine, seamless collaboration between health care professionals, such as physicians and nurses, is essential in achieving optimal patient care and a high level of patient safety [1-3]. This need is particularly pronounced in emergency medicine, where rapid and coordinated teamwork is critical to managing life-threatening situations [2]. Although teamwork is so important for patient outcomes, there is hardly any time to practice this during work [4], especially in the emergency department. One way to counteract this is to integrate interprofessional education (IPE) into the curriculum of health professionals [2,5-7]. IPE involves students from at least 2 different health care professions, who must learn to understand their respective roles and responsibilities and practice their communication skills and thereby enhance their ability to work seamlessly together in real emergency situations [4,7-10].

The importance of IPE during undergraduate studies is widely recognized, and efforts are underway worldwide to integrate this into health care education [4,11-13]. In Switzerland, for example, the Federal Law on Health Professions requires “familiarity with the interaction between different health professions” as a necessary expertise for graduates [14]. Consequently, Swiss nursing education has made progress in promoting interprofessional collaboration through joint educational activities involving diverse professions, such as midwives, nutritionists, and physiotherapists [15]. In Swiss medical education, the importance of interprofessional work has also been recognized, as is seen in its inclusion in the medical licensing examination. However, it has not been consistently integrated into the curriculum [16]. Previous interprofessional courses have faced challenges and some have been discontinued, in part due to the COVID-19 pandemic [17,18]. As a result, there remains a lack of interaction between nursing and medical students. Addressing this issue is critical given the lifelong collaboration between these professions in the medical field.

To foster interprofessional collaboration and enhance student teamwork skills, team training is an effective approach, as evidenced by numerous reviews [5-7,19]. Team training is commonly conducted through real-life simulations, “a technique...to replace or amplify real experiences with guided experiences, often immersive in nature, that evoke or replicate substantial aspects of the real world in a fully interactive fashion” [20]. Such simulations are frequently carried out in simulation centers, although these require substantial resources, and this limits their accessibility [6,21]. Virtual reality (VR)–based training is an innovative and resource-efficient alternative, which offers immersive experiences that simulate real-world scenarios [22,23]. Current research suggests that VR simulations are at least as effective as real-life simulations and offer advantages such as reduced costs, accessibility, and the ability to practice challenging scenarios [24-28].

Our objective was therefore to develop and evaluate an interprofessional team (INTEAM) training course for students that uses a VR simulation to provide them with an authentic training experience of handling an emergency case, coupled to interprofessional communication. We now report the development, content, and evaluation of the INTEAM training course “Patient handover and headache.” We specifically aimed to assess the acceptance (usability, VR-induced sickness, sense of presence, workload, user satisfaction, and technology acceptance) of the VR simulation among nursing and medical students, with comparisons between study programs and genders; its learning outcome among nursing and medical students, including comparisons between study programs; and its feasibility.

## Methods

### Study Design and Setting

In this evaluation study, a 1-group pre- and posttest design was used. Data collection took place in May 2023 digitally and in a training facility of the University Hospital of Bern, Switzerland.

Beyond the reported data, additional data on the validation of the TEAM (Team Emergency Assessment Measure) instrument [29] collected during the project was reported elsewhere [30]. The data presented in the 2 papers do not overlap, with the exception of demographic information.

### Ethical Considerations

The local ethics committee (Kantonale Ethikkommission Bern) deemed our study to be exempt from full ethical approval, as it is not covered by the Human Research Act (BASEC-Nr: Req-2023‐00208).

### Participants and Eligibility Criteria

The study population was a convenience sample of adult (≥18 years of age) final-year medical students of the University of Bern and adult (≥18 years of age) final-year bachelor nursing students of Bern University of Applied Sciences. The project was presented to them by their teachers. Students could volunteer for the course at their university (nursing) or enroll themselves through the course portal (medicine) as one of several optional courses in the spring semester. No compensation was provided. Written informed consent for study participation and publication of study results was obtained from each student.

Exclusion criteria included unwillingness to participate or give informed consent. Students experiencing epilepsy or other sensitivity to flashing light were also excluded.

### Training Course

#### Overview

The development of the INTEAM training course in emergency medicine involved experts from various disciplines, including emergency physicians, nurses, medical educators, and psychologists, who determined the learning objectives based on the entrustable professional activity (EPA) and designed the course content and material and the VR case scenario. Given the course’s focus on EPA 6—“Recognize a patient requiring urgent/emergency care, initiate evaluation and management” [16]—the team specifically selected a common emergency medical scenario that required seamless interprofessional collaboration, including 2 structured handovers.

To reduce the time needed for the on-site training, we decided to include an e-learning part that had to be accomplished at home in preparation of the on-site part. The e-learning part aimed at refreshing the major contents of the simulation (headache with red flags, epileptic seizure, and the handover tool “Introduction, Situation, Background, Assessment, and Recommendation” [ISBAR] [31-33]), which had already been covered by the respective curricula.

The on-site part was scheduled for 3 consecutive days, with each day consisting of 3 separate 3-hour time slots. Per slot, 6 students were invited (3 nursing and 3 medical students), as we had 3 rooms and 3 moderators available. Upon arrival, students were given a prebriefing and an orientation tour to introduce them to the upcoming VR simulation. In the VR simulation, the nursing and medical students had the opportunity to practice their future role as nurses or physicians, including their teamwork, as interprofessional pairs in a specific emergency situation. After the simulation, students participated in a debriefing to reflect on their experience and learning points.

#### e-Learning

Students received a link to the e-learning part approximately 1 week before the on-site session. The e-learning part consisted of 2 videos of 10 minutes each. In the first video, an emergency physician and an advanced practice nurse—with the support of a facilitator—gave an overview of headaches and epileptic seizures (ie, clinical manifestation, diagnostics, and therapy) and shared their best practices. In the second video, the same team presented the dos and don’ts of handovers and emphasized the ISBAR handover tool together with a typical demonstration. Students were also pointed to a written summary of the presentation, further examples, and background material.

#### VR Simulation

##### Development

The interdisciplinary author team developed the simulation case, with input from a company for immersive technologies in the health care sector (StellDirVor GmbH). The implementation in VR was carried out by the VR medical simulation company SimX.

The case displayed a male, 56-year-old patient initially presenting to the emergency department with a thunderclap headache and subtle neurological findings and then progressing to a generalized tonic-clonic epileptic seizure due to subarachnoid hemorrhage. The developers carefully considered the main learning objectives (following EPA 6): emergency management of a patient with thunderclap headache and epileptic seizure (including history taking); physical (including neurological) examination; use of the ABCDE (Airway, Breathing, Circulation, Disability, Exposure) approach [34] in the emergency setting; drug therapy; and conducting a structured handover using ISBAR.

##### Technical Details and Simulation Setup

The VR simulation setup consisted of the application of a fully immersive semiautomated supervised-learning VR training scenario using a Meta Quest 2 VR headset with Elite Straps with built-in battery, touch controllers (Meta Platforms, Inc), and noise-canceling headphones (JBL Tune 760NC). The VR simulation was controlled by a moderator using an OMEN Gaming Laptop by HP (HP Development Company), giving the appropriate prerecorded verbal responses and initiating the appropriate physiological patient response. There were 3 moderators (2 medical students and 1 PhD student with a background in nursing) and a coordinator (PhD student with a background in psychology) who received training from the study team for approximately 5 hours in the emergency scenario and the technical setup. We deliberately selected students as moderators, in concordance with the principles of peer-tutor teaching. The coordinator also served as a substitute moderator and was familiar with the VR system to help the moderators troubleshoot technical issues.

##### Prebriefing

At the beginning of the on-site part, we conducted a prebriefing with all 6 students to communicate the key aspects, including safety guidelines for the VR experience. We emphasized the importance of creating a safe learning environment and assured students that their performance would only be analyzed for study purposes and in pseudonymized form. We also made clear that the main aim of the course was not to assess their clinical skills but rather to explore the use of VR and an interprofessional course as an effective learning tool.

##### Orientation Tour

**Figure 1.**
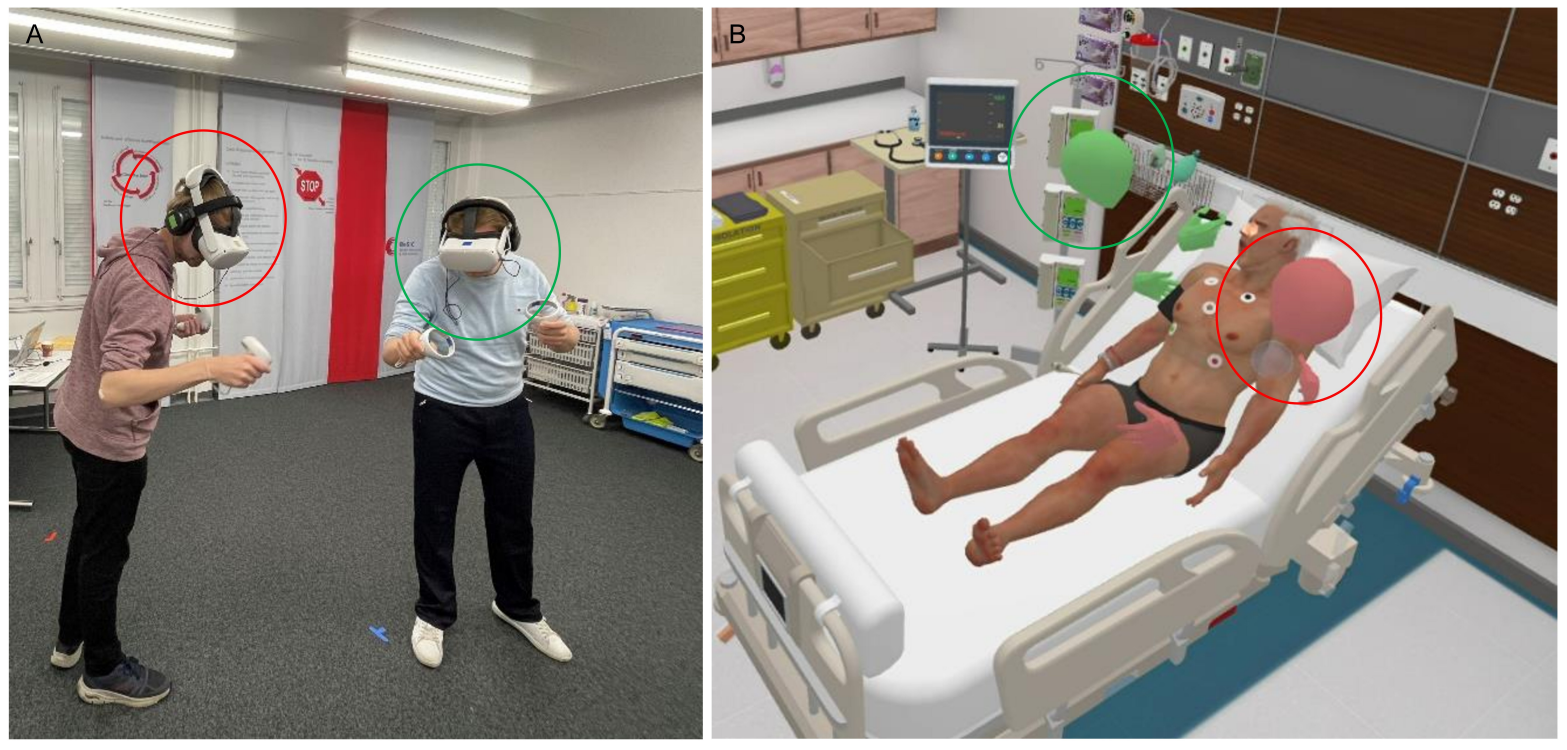
Participants in the VR simulation (**A**) in the real training room and (**B**) in the VR patient room. The circles illustrate the corresponding avatars. VR: virtual reality.

After the prebriefing, students were randomly assigned to pairs of a nursing and a medical student. Each pair was accompanied to a separate room by one of the moderators. Prior to the VR simulation, the student pairs were guided through the VR environment by the moderator in a standardized procedure. During this orientation tour, the team could familiarize themselves with their virtual surroundings and practice how to interact with the virtual environment. They were informed that their teammates’ avatars would just be displayed as heads and hands in VR (Figure 1) due to the technical setup of the simulation software. The students were also instructed to speak in standard German, without using dialects, but the patient would respond in English.

##### Content of VR Simulation

Before entering the virtual patient room, the nursing student received a brief written instruction from the moderator:

You are a nurse working in a local hospital. You are called to a room in the emergency department to see a new patient. The patient has walked in on his own and has not yet been seen by a physician. Please perform the initial assessment. The physician will soon come to support you. The physician on duty will knock on the door and then support you.

**Figure 2.**
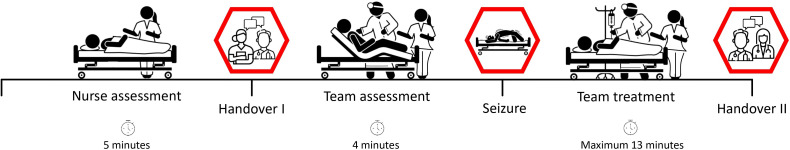
Virtual reality simulation scenario.

The student nurse then started to take the history and examination of the patient who presented with a severe headache and who was accompanied by his wife (nurse assessment, Figure 2). In the meantime, the medical student stayed in the same (physical) room, but was unable to watch or listen to the nurse-patient interaction, and also received written instructions:

You are a physician working in a regional hospital. You are called to see a new walk-in patient in the emergency department. The nurse is already there and asks for your assistance. If you hear the knock [of the moderator], you can enter and introduce yourself to the nurse. If you don't get a handover from the nurse, ask for one.

The medical student entered the VR simulation after 5 minutes, and the first handover from the nursing student to the medical student took place. The team was then able to continue taking the history and physical (including neurological) examination (team assessment). After 9 minutes (in total), the patient had an epileptic seizure, which required emergency care such as placing him in a stable side position and administering oxygen and medications. The seizure lasted until it was treated with benzodiazepine or anticonvulsants or ceased spontaneously without treatment at minute 17. After the seizure, the patient was unresponsive for 3 minutes (postictal), after which his cognitive state gradually improved. During this phase (team treatment), the students were able to order further diagnostics and call the attending physician. The VR simulation ended with either a self-initiated handover to the attending physician or a moderator-triggered handover at minute 23 via telephone.

### Debriefing

The 30-minute lasting debriefing was conducted after the VR simulation by the moderators according to a debriefing guideline. The guideline was developed from an expert in simulation training (TCS) following the 3D Model of Debriefing [35] and included 4 subjects: defusing (How did it go? How did it feel?); discovering, including medical aspects (What was the medical problem? What did you do?); handover (Did you get all the information? What were the problems?); and deepening (What have you learned for the interprofessional teamwork? What will you take with you to the next emergency handover?). The debriefing session was neither recorded nor analyzed.

### Data Collection

#### Overview

Before and after the VR simulation, we administered questionnaires as outlined in Table 1. The self-constructed questionnaires can be found in Multimedia Appendix 1.

**Table 1.** Overview of all questionnaires.

Category and variable measured		Questionnaire	Point in time
**Baseline**			
	Age, gender, study program, prior medical education (eg, health care assistant), previous experience with VR^a^ simulation and gaming, previous communication training	Self-constructed questionnaire (digital)	Baseline (approximately 2 weeks before the on-site part)
	Visual aid	Self-constructed questionnaire	Presimulation
	Acquaintance	Self-constructed questionnaire	Postsimulation
**Acceptance**			
	Usability	System Usability Scale [36]	Postsimulation
	VR-induced sickness	Simulator Sickness Questionnaire [37]	Postsimulation
	Sense of presence	Slater, Usoh, and Steed [38]	Postsimulation
	Subjective workload	NASA-Task Load Index [39]	Postsimulation
	User satisfaction	User Satisfaction Evaluation Questionnaire [40]	Postsimulation
	Technology acceptance	Fast Form-Technology Acceptance Model [41]	Postsimulation
**Learning outcomes**			
	Handover skills	Clinical case vignette 1 and 2 (digital) and rated with Handover Assessment Tool [42]	Baseline and postdebriefing
	Confidence	Self-constructed questionnaire	Baseline and postdebriefing
	Perceived effectiveness	Training Evaluation Inventory [43]	Postsimulation
**Feasibility**			
	Duration, technical problems, attendance of on-site part	Self-constructed questionnaire (moderator)	On-site part
	Evaluation of the e-learning, VR simulation, and debriefing regarding the achievement of the learning objectives, grade, and suggestions for improvement	Self-constructed questionnaire	Postdebriefing
	Overall comments (regarding feasibility)	Free text	Postdebriefing

a VR: virtual reality.

#### Baseline Data

All students had to fill in a web-based questionnaire via SosciSurvey before starting the INTEAM training course. The link to the questionnaire was sent to students approximately 2 weeks before the on-site session and 1 week before the e-learning. It included questions about sociodemographic factors (age, gender, study program, and prior medical education), previous experience with VR simulation and gaming, and previous communication training (in hours). The use of visual aids was assessed in a survey right before the VR simulation. The students were also asked whether they knew their assigned team partner.

#### Acceptance

The following questionnaires were completed by the students immediately after the VR simulation.

Usability was assessed using the System Usability Scale (SUS) [36], which consisted of 10 questions to be rated on a 5-point Likert scale, ranging from 1=strongly disagree to 5=strongly agree. Ratings were then converted so that the resulting SUS score ranges from 0 to 100, with scores lower than 50 being regarded as concerning [36,44].

VR-induced sickness was assessed with the Simulator Sickness Questionnaire (SSQ) [37], where the students had to rate 16 symptoms (eg, nausea and headache) on a 4-point Likert scale from 0=none to 3=severe. Ratings were then converted into 3 subscores and a total score. A total SSQ score above 20 is an indicator of a poor simulator according to Stanney et al [45].

Sense of presence in the virtual world was assessed using the 6-item questionnaire developed by Slater, Usoh, and Steed [38,46], using a semantic differential scale (eg, 1=being elsewhere and 7=sense of being in the virtual environment), with a mean score of 7 representing the strongest sense of presence.

Perceived subjective workload was assessed using the NASA (National Aeronautics and Space Administration)–Task Load Index (NASA-TLX) [39]. The NASA-TLX was calculated by weighting 6 dimensions (mental demand, physical demand, temporal demand, performance, effort, and frustration) assigned by the respondent, with each dimension’s rating, and summing these weighted values. The calculated total score can range from 0 to 100, with higher scores indicating more perceived subjective workload.

User satisfaction was assessed using the User Satisfaction Evaluation Questionnaire [40], comprising 6 questions with a 5-point Likert scale from 1=not at all to 5=very much. The total score can range from 6=poor satisfaction to 30=excellent satisfaction.

Technology acceptance was measured using the Fast Form-Technology Acceptance Model (FF-TAM) [41,47], comprising 12 items scored on a 7-point semantic differential scale (eg, 1=ineffective and 7=effective). The FF-TAM mean score ranges from 1 to 7. Higher scores reflect an increased likelihood of technology acceptance based on the subscales usefulness (items 1‐6) and ease of use (items 7‐12).

#### Learning Outcome

As one of the learning objectives was to practice a structured handover using ISBAR, handover skills were assessed with a pre- and posttest to assess the training’s learning outcome. The pretest took place as part of the web-based questionnaire mentioned earlier prior to the commencement of the e-learning. For this, students were given 4 minutes to read a clinical case vignette (case 1) and take notes. The screen then automatically showed the next page, where they were instructed to record a verbal, structured handover of the case within 1 minute. The posttest took place after the debriefing, following the same procedure as in the pretest, now with a different clinical case vignette (case 2; Multimedia Appendix 2). Both handovers were transcribed and assessed by 1 trained rater (ANN) using the Handover Assessment Tool (HAT) [42], which was adapted for our study purposes (Multimedia Appendix 3). The tool comprises 12 items and follows the ISBAR framework, resulting in a total score of 0‐12 points, with higher values indicating better adherence to the ISBAR framework. To capture the perceived learning outcome, confidence was assessed at baseline in the web-based survey and after the INTEAM training course using a self-constructed 4-item questionnaire (“rate your confidence when (1) caring for a patient with a seizure, (2) making a structured handover of an emergency patient, (3) recognizing when to call for help in an emergency situation, and (4) working with a person from another profession”) on a 5-point Likert scale (1=very low to 5=very high). The confidence items were based on items from Kolbe et al [12] and have already been tested in a study by Birrenbach et al [48] as well as in the pilot.

In addition, the perceived effectiveness of the VR simulation was measured immediately after the simulation using the Training Evaluation Inventory [43], which consists of 17 statements regarding 5 subscores: subjective enjoyment, perceived usefulness, perceived difficulty, subjective knowledge gain, and attitude toward the training, scored on a 5-point Likert scale ranging from 1=strongly disagree to 5=strongly agree.

#### Feasibility

To assess the feasibility of the training, notes were taken by the moderator on the duration of the orientation and the VR simulation as well as on any technical problems or other comments regarding the on-site part. The notes of all training were summarized and analyzed descriptively.

The students also evaluated the e-learning, the VR simulation, and the debriefing separately after the debriefing session by answering 3 questions: “How well did this part contribute to achieving the learning objectives?” (1=not at all to 6=very well), “What grade would you give this part?” (1=worst to 6=best), and “Do you have any suggestions for improvement?” (free text). Students were also asked to write down their most important learning experience during the course (free text). There was also space for overall comments regarding feasibility (free text). The free-text responses were coded by one of the authors (ANN), then summarized, and analyzed descriptively.

### Statistical Analysis

The data were analyzed using SPSS (version 28.0; IBM Corp) and stored in pseudonymized form. Only the data of complete teams were analyzed. Descriptive statistics, the Mann-Whitney *U* test, and the Fisher exact test were used to compare the baseline characteristics between groups.

Wilcoxon signed rank tests were performed for pre- and postsimulation comparisons (ie, handover skills and confidence). The Mann-Whitney *U* test was used to compare the questionnaire results of medical with those of nursing students and of women with those of men. A *P* value of <.05 was considered statistically significant.

## Results

### Overview

**Figure 3.**
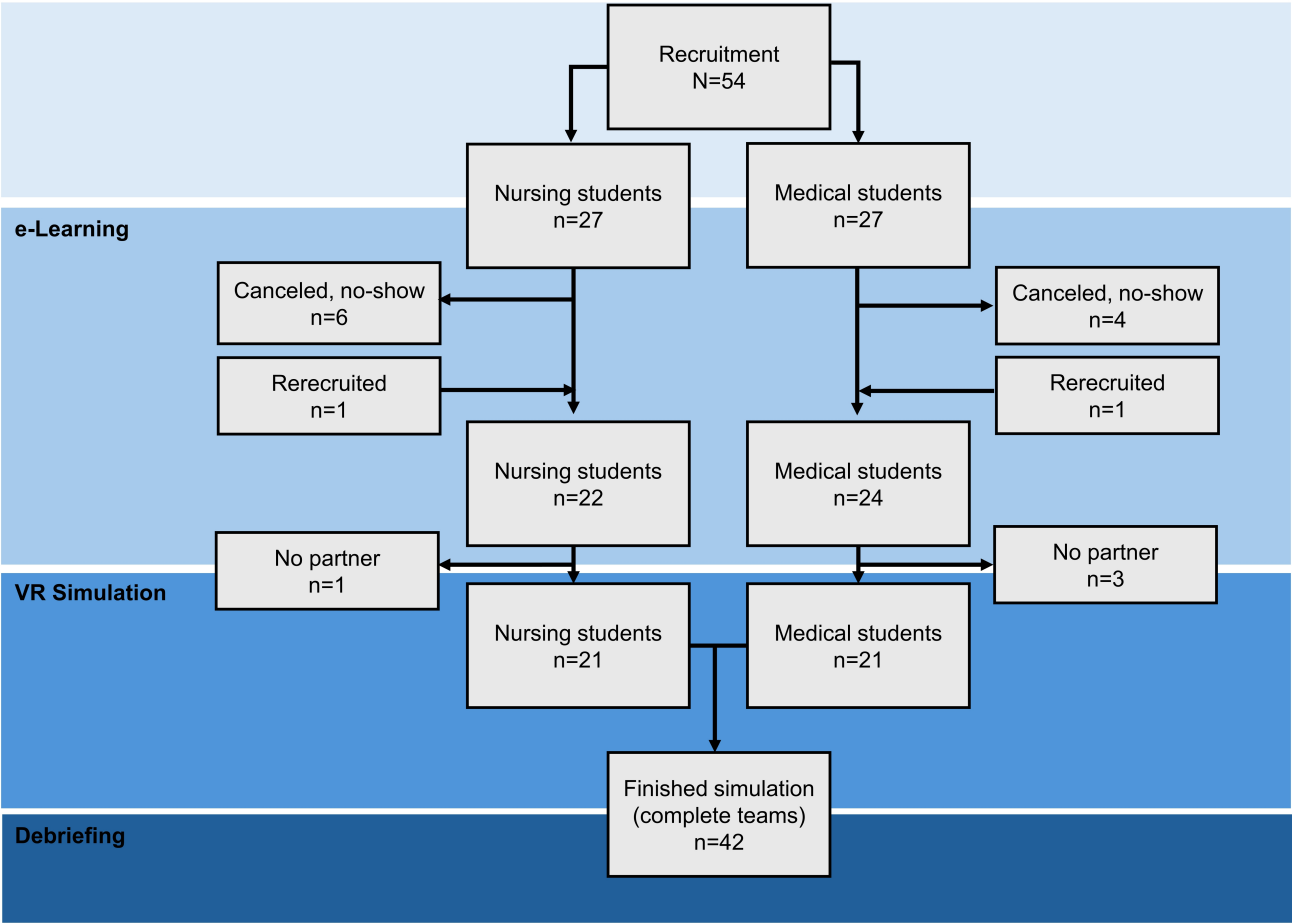
Flowchart. VR: virtual reality.

As shown in Figure 3, 54 students enrolled in the course. Among them, 46 completed the e-learning part. Due to no-shows, 4 students had no team partner for the VR simulation. In these cases, the nonpaired students were still invited to conduct the simulation in a modified manner (shorter, moderator as a partner) but excluded from the evaluation. The final sample thus comprised 42 students, including 21 nursing and 21 medical students.

### Sample Characteristics

Table 2 shows the demographic characteristics of our final sample. One nursing student did not complete the baseline questionnaire, but gender and course of study were used, as this was known. Only 1 team was familiar with each other; they had known each other for 6 years. Nursing students differed from medical students in terms of age, prior medical education, and hours of communication training, which may be explained by the different lengths of their curricula (nursing 3 years and medicine 6 years).

**Table 2.** Baseline characteristics.

Characteristics	Nursing students (n=20)	Medical students (n=21)	Total (n=41)	*P* value
Age (years), median (IQR)	23 (22‐24)	26 (25‐28.5)	25 (23‐26)	*<.001^a^* ^,^ ^b^
Female, n (%)	16 (76)^c^	10 (48)	26 (62)^d^	.06^a^
**Visual aids, n (%)**				.48^a^
Glasses	5 (24)^c^	6 (30)^e^	11 (27)	
Lenses	3 (14)^c^	4 (20)^e^	7 (17)	
Communication training (hours), median (IQR)	8.5 (7‐18.8)	15 (12‐25)	14 (8.5‐20)	*.006* ^a^
Prior medical education, n (%)	6 (30)	0 (0)	6 (15)	*.007* ^a^
**Playing computer games, n (%)**				.13^f^
Several times a week	2 (10)	2 (10)	4 (10)	
About weekly	1 (5)	4 (19)	5 (12)	
1‐2 times per month	4 (20)	0 (0)	4 (10)	
1‐2 times per year	1 (5)	5 (24)	6 (15)	
Less than 1‐2 times per year	3 (15)	2 (10)	5 (12)	
Never	9 (45)	8 (38)	17 (42)	
**Virtual reality simulations, n (%)**				.44^f^
Several times a week	0 (0)	0 (0)	0 (0)	
About weekly	0 (0)	0 (0)	0 (0)	
1‐2 times per month	1 (5)	0 (0)	1 (2)	
1‐2 times per year	0 (0)	1 (5)	1 (2)	
Less than 1‐2 times per year	4 (20)	7 (33)	11 (27)	
Never	15 (75)	13 (62)	28 (68)	

a Mann-Whitney *U* test.

b Significant values are present in italics format.

c n=21.

d n=42.

e n=20.

f Fisher exact test.

### Acceptance

To quantify the acceptance of the VR simulation, we report the median scores of the usability, VR-induced sickness, sense of presence, subjective workload, user satisfaction, and technology acceptance scales (Table 3).

**Table 3.** Acceptance variables.

	Nursing students (n=21), median (IQR)	Medical students (n=21), median (IQR)	Total (n=42), median (IQR)	*P* value^a^
**Usability: SUS^b^ (score ranges 0 to 100, higher=better)**				
Total score	70 (65‐81.3)	77.5 (67.5‐80)	72.5 (65-80)	.34
**Virtual reality–induced sickness: SSQ^c^ (total score ranges 0 to 236, higher=more symptoms)**				
Total score	30 (11‐43)	26 (13‐37)	26 (11‐41)	.34
Nausea	19.1 (9.5‐28.6)	19.1 (0‐28.6)	19.1 (9.5‐28.6)	.11
Oculomotor	45.5 (15.2‐60.6)	37.9 (15.2‐60.6)	37.9 (15.2‐60.6)	.99
Disorientation	41.8 (20.9‐83.5)	41.8 (27.8‐62.6)	41.8 (27.8‐73.1)	.66
**Sense of presence: Slater, Usoh, and Steed (score ranges 0 to 7, 7=strongest sense of presence)**				
Total score	5 (4.3‐5.4)	4.5 (3.9‐5.5)	4.8 (4.1‐5.5)	.86
**Subjective workload: NASA-TLX^d^ (score ranges 0 to 100, higher=more workload)**				
Total score	64.3 (60‐69.2)	64.7 (55.2‐71.3)	64.5 (59.6‐70.7)	.89
**User satisfaction: USEQ^e^ (score ranges from 6=poor satisfaction to 30=excellent satisfaction)**				
Total score	24 (21.5‐28)	25 (23‐27)	25 (22.8‐27)	.93
**Technology acceptance: FF-TAM^f^ (score ranges from 1 to 7, higher=better)**				
**Subscore**				
Usefulness	6.3 (5.8‐6.8)	6 (5.5‐6.5)	6 (5.8‐6.9)	.78
Ease of use	6 (4.7‐6.3)	5.8 (5.3‐6.3)	5.9 (5.1‐6.3)	.49

a Mann-Whitney *U* test.

b SUS: System Usability Scale.

c SSQ: Simulator Sickness Questionnaire.

d NASA-TLX: NASA-Task Load Index.

e USEQ: User Satisfaction Evaluation Questionnaire.

f FF-TAM: Fast Form-Technology Acceptance Model.

Usability was rated as good (SUS: median 72.5, IQR 65-80 of a maximum of 100). SSQ analysis showed moderate VR-induced sickness, primarily due to eye-related problems (eg, difficulty focusing and blurred vision). Perceived participant presence (Slater, Usoh, and Steed) was good, with a median rating of 4.8 (IQR 4.1-5.5) of a possible 7. Students also expressed positive satisfaction (User Satisfaction Evaluation Questionnaire) with the system, giving a median score of 25 (IQR 22.8-27) of a maximum 30. Moreover, technology acceptance (FF-TAM) was deemed satisfactory, with a usefulness median of 6 (IQR 5.8-6.9), and an ease of use median of 5.9 (IQR 5.1-6.3) of a possible 7. However, the workload was rated as notably high, as evidenced by a NASA-TLX median score of 64.5 (IQR 59.6-70.7) of 100.

As can be seen in the last column of Table 3, there were no significant differences between nursing and medical students (all *P*≥.11). However, there were significant differences between female (median 71.3, IQR 62.5‐80) and male students (median 78.8, IQR 70.6‐86.3) in the total score of the SUS, with male students rating usability higher (*z*=−2.380; *P=*.02; *r*=0.37). Additionally, female students (median 35.5, IQR 20.3‐49.8) had significantly higher scores than male students (median 20.5, IQR 8‐26) in the total score of the SSQ (*z*=−2.586; *P=*.01; *r*=0.39) and its subscore disorientation (median 62.6, IQR 27.8‐90.5 and median 34.8, IQR 13.9‐52.2, respectively; *z*=−2.481; *P=*.01; *r*=0.38).

### Learning Outcome

For 36 students, we obtained complete recordings of the pre- and posttests on handover skills, which were evaluated with the HAT [42]. The recordings of 5 nursing students and 1 medical student could not be evaluated due to recording errors (blank recording, recording canceled, and recording not executed). Results indicated that nursing students had a lower pretest score than medical students (median 7, IQR 5‐8 and median 8, IQR 7‐9, respectively; *z*=−1.93; *P=*.045; *r*=0.33). The HAT score (all students) showed a significant increase from the pretest (median 8, IQR 6‐9) to posttest (median 8, IQR 7‐9; *z*=−2.01; *P*=.045; *r*=0.33).

For 41 students, we obtained complete pre- and postdebriefing confidence ratings (1 student did not fill out the pretest). At pretest, students rated their confidence as medium to high (Table 4). For 2 items (caring for patient and call for help), ratings improved at posttest but not for the other 2 (*z*=−3.8; *P*<.001; *r*=0.60 and *z*=−3.0; *P=*.003; *r*=0.47, respectively). Interestingly, medical students indicated lower confidence than nursing students at pretest for the item “working interprofessionally” (*z*=−2.3; *P=*.02; *r*=0.36).

**Table 4.** Confidence.^a^

Confidence: items	Nursing students (n=20)			Medical students (n=21)			Total (n=41)		
	Pre, median (IQR)	Post, median (IQR)	*P* value^b^	Pre, median (IQR)	Post, median (IQR)	*P* value^b^	Pre, median (IQR)	Post, median (IQR)	*P* value^b^
Caring for patient with seizure	3 (2‐3.8)	3 (3-4)	.06	3 (2-3)	4 (3-4)	*<.001^c^*	3 (2-3)	3.5 (3-4)	*<.001*
Making structured handover	3 (3-4)	3 (3-4)	.26	3 (3-4)	3 (3-4)	>.99	3 (3-4)	3 (3-4)	.47
Recognizing when to call for help	4 (3-4)	4 (4-4)	*.03*	4 (3-4)	4 (4-4)	*.03*	4 (3-4)	4 (4-4)	*.003*
Working interprofessionally	4 (4‐4.8)	4 (4-4)	.25	4 (3-4)	4 (3-4)	.36	4 (4-4)	4 (4-4)	.87

a Likert scale from 1=very low to 5=very high.

b Wilcoxon signed rank test.

c Significant values are present in italics format.

The results of the Training Evaluation Inventory indicate a high perceived effectiveness. The subscores subjective enjoyment, perceived usefulness, and attitudes toward training were rated high (median 5, IQR 4‐5). The subscores perceived difficulty and subjective knowledge gain received medium ratings (median 4, IQR 4‐5 and median 4, IQR 3‐5, respectively). No significant differences were found between nursing and medical students (all *P*≥.25).

### Feasibility

The mean length of the orientation tour was 26 (SD 6) minutes, and that of the VR simulation part was 20 (SD 4) minutes. In nearly 67% (n=14) of the 21 VR simulations, technical difficulties arose, but all simulations could be completed. The most serious problems were hardware and software related and involved tracking, resulting in incorrect spawn or disconnection from the VR simulation. There was also 1 case of continuous controller vibration. Software-related problems included the patient overstretching his head and not speaking when the hand (or the controller) came too close to his neck. In addition, some VR simulation materials that had been placed on the patient, such as the blood pressure cuff, were no longer attached to the patient after switching to another phase of the simulation (eg, postictal). There were also situations where either the patient’s responses were no longer heard by both students or 1 student could no longer hear the other student and the patient. Other issues were reported, but they were not related to technical problems, like blurred vision or double hearing. Students heard the team member through noise-canceling headphones due to the microphone setup (for recording), but also heard others in real time, causing a confusing mix of delayed and overlapping sounds.

Analyses of the questionnaires revealed that students rated both the e-learning and VR simulation parts as contributing well to achieving the learning objectives (median 5, IQR 4‐5). They also gave the same high grade to both parts (median 5, IQR 4‐5). The debriefing received similarly high ratings (median 5, IQR 4.8‐6) and grades (median 5, IQR 5‐6).

The coding system and some examples of the free-text responses can be found in Multimedia Appendix 4. Overall, there were only a few suggestions for improvement. Suggestions were made on improving the e-learning content, some of which were inconsistent, such as indicating that the e-learning was overloaded versus that it should be expanded. Feedback on the VR simulation pointed to technical limitations and the need for a longer duration. Suggestions for debriefing related to content and structure. Key learning experiences included interprofessional working, teamwork, handover, and the effectiveness of VR as a learning tool. The overall comments covered a range of issues, with some mentioning technical distractions and a preference for more human-like avatars. Some students found the VR experience new and stressful and suggested repetition for better adaptation. Students emphasized the importance of the orientation tour and the need for clear instructions. Again, some students mentioned the duration of the simulation (too short) and that the VR was a valuable learning tool. Some specifically highlighted its effectiveness for team training and the acquisition of standards.

## Discussion

### Principal Findings

This evaluation study examined the acceptance, learning outcomes, and feasibility of the newly developed INTEAM training course, in which 21 pairs of nursing and medical students trained together their medical and handover skills in a VR simulation. Results of questionnaire analyses and pre- and posttest comparisons indicate that the course was perceived as highly acceptable, feasible, and may be effective in improving handover skills. In the following, we summarize and discuss the principal findings.

### Acceptance

The acceptance of the VR simulation as part of the INTEAM training course was measured by usability, VR-induced sickness, sense of presence, subjective workload, user satisfaction, and technology acceptance. The INTEAM training course demonstrated good usability [44], despite a relatively high rating for VR-induced sickness using the SSQ [37]. Previous research suggests that high levels of simulator or VR-induced sickness can lead to reduced usability [49,50]. However, it is possible that the SSQ is not the most appropriate tool for capturing usability in a VR setting, as cybersickness can differ from simulator sickness [45]. Instead, the use of a specific tool such as the Virtual Reality Sickness Questionnaire may provide more accurate results [51]. In both measures, VR-induced sickness and usability, we observed a gender difference, namely that men rated usability higher and reported less VR-induced sickness, and this is consistent with findings from other studies [52,53].

Compared to other studies [54], the workload was judged as fairly high, potentially due to most students being in a VR simulation for the first time. Although some research indicates a lower workload in VR [28], this pertained to single-player skill training without time constraints. Conversely, other studies have noted increased workload under stress [54,55]. Students mentioned that they had felt additional stress due to the unfamiliarity of the VR tool, particularly during the already tense emergency simulation. Nonetheless, despite these challenges, students expressed a high level of satisfaction.

### Learning Outcomes

The learning outcomes of the INTEAM training course were assessed through students’ performance in structured handover, self-reported confidence level, and their evaluation of the effectiveness of the training. When comparing handover quality before and after the training, we observed a slight improvement in ISBAR-related learning, which is consistent with studies that showed enhanced outcomes when ISBAR training is coupled with simulations rather than solely relying on theoretical instruction [42,56].

At baseline, medical students showed less confidence in interprofessional work than nursing students possibly because the latter had attended more interdisciplinary lectures on various topics. Students’ confidence in handling seizure cases increased significantly after the training, probably due to the integration of theory and practice in an authentic yet controlled environment [26]. The positive comments about VR as a learning tool support the assumption that VR is particularly effective for Generation Z students [57].

### Feasibility

Feasibility was captured by recording technical aspects such as the duration of the orientation and VR simulation session, any technical problems, and comments from the moderator. In terms of content, students evaluated e-learning, VR simulation, and debriefing using predefined questions and shared their main learning experiences.

We experienced technical difficulties, but most of them were easily resolved with minimal effort. None of the VR simulations had to be terminated due to technical malfunctions. This may be attributed to the presence of a coordinator who was always available and had high technical expertise. Some disturbances, like blurred vision, were attributed to slight movements of the head-mounted displays, although the Elite Strap helped secure them; nevertheless, text in VR was acknowledged as being potentially less clear than in reality. The additional battery in the Elite Strap proved beneficial and ensured that there were no battery-related interruptions during the training sessions.

One issue with the feasibility was the absence of any replacements who could take over if a student failed to appear. This is also a crucial consideration when incorporating such training into a curriculum [58]. There will typically be a larger number of students, and they will need to be divided into smaller groups. One potential approach is to involve several students per session, with some observing while others participate, as they did in other studies [59]. This facilitates a peer-tutor dynamic while ensuring that at least 1 nursing and 1 medical student are present, even if attendance is not consistent.

As indicated by the grading of the participants, the course content seems feasible. Even though there were a few improvement suggestions, some of these were inconsistent. The positive ratings of the learning objectives by both groups lead us to believe that we have successfully developed valuable learning objectives that are not only interprofessional but also individually relevant for each group.

Students expressed positivity toward the interprofessional approach and would appreciate the inclusion of interprofessional training in their curricula. Other studies showed similar findings [60,61].

### Strengths and Limitations

The study’s key strengths include the development and evaluation of the INTEAM training by an interdisciplinary team comprising experts in medical education, emergency medicine, psychology, and nursing. This ensured a comprehensive and well-rounded approach to the project. Furthermore, the study used validated and widely used tools for assessing VR-specific outcomes, enhancing the reliability and relevance of the findings. The study also adhered to methodological rigor, contributing to the robustness of the results. However, this study has some limitations worth noting. First, due to the study design, such as the absence of a control group and the relatively small sample size, the statistical robustness and generalizability of the results may be affected. While the findings offer valuable initial insights, it is important to note that no causal relationships can be inferred. Second, voluntary student participation might have introduced self-selection bias. Third, the assessment of handover quality as an indicator of learning outcome relied on a single evaluator and thus may have introduced observer bias despite the evaluator’s extensive practical experience. Fourth, not varying the order of vignettes between pre- and posttest of handover quality might have limited the assessment’s sensitivity. However, the second vignette was recognized by experts as the more challenging one, which further supports the results that suggest an increase in handover performance.

### Future Directions

We believe that there should be careful consideration and further research on the implementation of interprofessional training courses that include a VR simulation. It would be beneficial to investigate whether the technical aspects become less prominent when the VR simulation is repeatedly practiced. Once improved software and hardware become available, it will, of course, be worth exploring whether this leads to overall improvements.

Furthermore, different INTEAM training courses should be developed to explore all the learning objectives that can be achieved through such training. In this case, it is essential to select learning objectives that are significant for all involved students such as, in our case, medical and nursing students. Moreover, we need further research that investigates long-term effects and whether students can strengthen their teamwork skills through these courses and derive practical benefits for their future professional lives, as is the case with traditional team training [10].

### Conclusions

The INTEAM training course, including a VR simulation in emergency medicine, was well received by the nursing and medical students, and their handover skills and confidence in managing patients with seizure were improved after the course. Although some technical problems occurred during the VR simulations, none resulted in dropout, thus confirming the feasibility of the approach. Technical enhancements and organizational considerations are advisable for further improvement. These promising results encourage the use of VR simulations for team training in the education of nursing and medical students.

## Supplementary material

10.2196/57117 Multimedia Appendix 1 Self-constructed questionnaires.

10.2196/57117 Multimedia Appendix 2 Clinical case vignettes.

10.2196/57117 Multimedia Appendix 3 Adapted Handover Assessment Tool.

10.2196/57117 Multimedia Appendix 4 Code system—gives further information about the code system from the free text questions and comments.

## Abbreviations

ABCDE: Airway, Breathing, Circulation, Disability, Exposure
EPA: entrustable professional activity
FF-TAM: Fast Form-Technology Acceptance Model
HAT: Handover Assessment Tool
INTEAM: interprofessional team
IPE: interprofessional education
ISBAR: Introduction, Situation, Background, Assessment, and Recommendation
NASA: National Aeronautics and Space Administration
NASA-TLX: NASA-Task Load Index
SSQ: Simulator Sickness Questionnaire
SUS: System Usability Scale
TEAM: Team Emergency Assessment Measure
VR: virtual reality
