# Supplementary material for: Interprofessional Team Training With Virtual Reality: Acceptance, Learning Outcome, and Feasibility Evaluation Study
Source: JMIR Serious Games. 2024 Nov 4;12:e57117. doi: 10.2196/57117 (PMC11554288; doi:10.2196/57117)
Supplement: Multimedia Appendix 1 [file games-v12-e57117-s001.docx]

**Table S1. Self-constructed items in German (original wording).**

|  | **Items** | **Values** |
| --- | --- | --- |
| **Baseline survey** | | |
|  | Geschlecht? | - Weiblich - Männlich - Divers |
|  | Alter? | Ich bin …. Jahre alt. |
|  | Studiengang? | - Pflege - Medizin |
|  | Hast Du eine oder mehrere vorangehende Berufslehren oder berufsqualifizierende Ausbildungen im medizinischen Bereich? | - Nein - Ja: Welche? Ausbildung(en) inkl. Jahre |
|  | Vorangehende Erfahrungen:   - Ich spiele Computerspiele. - Ich bin in VR-Simulationen | 1 mehrmals pro Woche – 6 nie |
|  | Wie viele Stunden an Kommunikationstrainings hast Du bisher ungefähr besucht? Inklusive den Trainings im Rahmen Deines Studiums. | Freitext |
|  | Benutzt du heute eine Sehhilfe? | - Brille - Kontaktlinsen - Nein |
|  | Hast Du Dein heutiges Teammitglied vorher schon gekannt? | - Nein - Ja. Seit … Monaten |
| **Confidence** | | |
|  | Wie beurteilst Du Deine Fähigkeit, eine*n Patient*in mit einem Krampfanfall zu versorgen? | 1 sehr gering - 5 sehr gut |
|  | Wie beurteilst Du Deine Fähigkeit, eine strukturierte Übergabe eines Notfallpatienten/einer Notfallpatientin zu machen? | 1 sehr gering - 5 sehr gut |
|  | Wie beurteilst Du Deine Fähigkeit, in einer Notfallsituation zu erkennen, wann Hilfe zu holen ist? | 1 sehr gering - 5 sehr gut |
|  | Wie beurteilst Du Deine Fähigkeit mit einer Person einer anderen Profession (d.h. Pflegefachkraft/Mediziner*in) zusammenzuarbeiten? | 1 sehr gering - 5 sehr gut |
| **Duration, technical problems, attendance of on-site part (Moderator)** | | |
|  | Technische Probleme? | - Nein - Ja. Welche? |
|  | Startzeiten | - Startzeit Room Tour - Startzeit VR Sim |
|  | Stoppzeiten | - Ende Room Tour - Ende VR Sim |
|  | Kommentare |  |
| **Evaluation of the e-learning, VR simulation, and debriefing regarding achievement of the learning objectives, grade and suggestions for improvement** | | |
|  | **1. Teil: eLearning-Einheit zu Kopfschmerz, Epilepsie und strukturierter Übergabe**  Lernziel: Grundlagen zu Kopfschmerz, Epilepsie und strukturierter Übergabe mittels ISBAR auffrischen und vertiefen | |
|  | Wie gut hat dieser Teil dazu beigetragen, das Lernziel zu erreichen? | 1 gar nicht – 6 sehr gut |
|  | Welche Note würdest du diesem Teil geben? | 1 schlechteste – 6 beste |
|  | Hast du Anmerkungen / Verbesserungsvorschläge für diesen Teil? | Freitext |
|  | **2. Teil: VR-Simulation**  Lernziele: Einen Patienten mit Epilepsie versorgen, eine strukturierte Übergabe mittels ISBAR machen. | |
|  | Wie gut hat dieser Teil dazu beigetragen, das Lernziel zu erreichen? | 1 gar nicht – 6 sehr gut |
|  | Welche Note würdest du diesem Teil geben? | 1 schlechteste – 6 beste |
|  | Hast du Anmerkungen / Verbesserungsvorschläge für diesen Teil? | Freitext |
|  | **3. Teil: Debriefing (Nachbesprechung in der Gruppe)**  Lernziel: Erfahrungen während der Simulation reflektieren, Fragen klären, Wissen vertiefen. | |
|  | Wie gut hat dieser Teil dazu beigetragen, das Lernziel zu erreichen? | 1 gar nicht – 6 sehr gut |
|  | Welche Note würdest du diesem Teil geben? | 1 schlechteste – 6 beste |
|  | Hast du Anmerkungen / Verbesserungsvorschläge für diesen Teil? | Freitext |
|  | Was war während des gesamten Trainings deine wichtigste Lernerfahrung? | Freitext |
|  | Kommentare | Freitext |

**Table S2. Self-constructed items in English.**

|  | **Items** | **Values** |
| --- | --- | --- |
| **Baseline survey** | | |
|  | Gender | - Female - Male - diverse |
|  | Age | I am … years old. |
|  | Study program | - Nursing - Medicine |
|  | Do you have one or more previous apprenticeships or professional qualifications in the medical field? | - No - Yes: Which? Training(s) incl. years |
|  | Previous experience:   - I play computer games. - I am into VR simulations. | 1 several times a week - 6 never |
|  | Approximately how many hours of communication training have you attended so far? Including the training sessions as part of your studies. | Free text |
|  | Do you use a visual aid today? | - Glasses - Contact lenses - No |
|  | Did you know your current team member before? | - No - Yes, for … month |
| **Confidence** | | |
|  | Rate your confidence when caring for a patient with a seizure. | 1 very low - 5 very high |
|  | Rate your confidence making a structured handover of an emergency patient. | 1 very low - 5 very high |
|  | Rate your confidence recognizing when to call for help in an emergency situation. | 1 very low - 5 very high |
|  | Rate your confidence working with a person from another profession. | 1 very low - 5 very high |
| **Duration, technical problems, attendance of on-site part (Moderator)** | | |
|  | Technical problems | - No - Yes. Which? |
|  | Start times | - Start time Room Tour - Start time VR Sim |
|  | End times | - End time Room Tour - End time VR Sim |
|  | Comments |  |
| **Evaluation of the e-learning, VR simulation, and debriefing regarding achievement of the learning objectives, grade and suggestions for improvement** | | |
|  | **Part 1: eLearning unit on headache, epilepsy and structured handover**  Learning objective: Refresh and deepen the basics of headache, epilepsy and structured handover using ISBAR | |
|  | How well did this part contribute to achieving the learning objectives? | 1 not at all – 6 very well |
|  | What grade would you give this part? | 1 worst – 6 best |
|  | Do you have any suggestions for improvement? | Free text |
|  | **Part 2: VR simulation**  Learning objective: To care for a patient with epilepsy, make a structured handover using ISBAR. | |
|  | How well did this part contribute to achieving the learning objectives? | 1 not at all – 6 very well |
|  | What grade would you give this part? | 1 worst – 6 best |
|  | Do you have any suggestions for improvement? | Free text |
|  | **Part 3: Debriefing**  Learning objective: Reflect on experiences during the simulation, clarify questions, deepen knowledge. | |
|  | How well did this part contribute to achieving the learning objectives? | 1 not at all – 6 very well |
|  | What grade would you give this part? | 1 worst – 6 best |
|  | Do you have any suggestions for improvement? | Free text |
|  | What was your most important learning experience during the course? | Free text |
|  | Comments | Free text |
