# Supplementary material for: Interprofessional Team Training With Virtual Reality: Acceptance, Learning Outcome, and Feasibility Evaluation Study
Source: JMIR Serious Games. 2024 Nov 4;12:e57117. doi: 10.2196/57117 (PMC11554288; doi:10.2196/57117)
Supplement: Multimedia Appendix 2 [file games-v12-e57117-s002.docx]

**Clinical Case Vignette 1**

You work in a small hospital in the emergency room. It is Friday towards evening, pre-Christmas and cold. The waiting room is very full and everyone is stressed. A 46-year-old man, Mr. Meier, comes alone to the emergency room. He has stabbing pains on the left side of his chest, feels generally weak. In addition, he gets out of breath quickly when climbing stairs. The pain started yesterday at dinner when he swallowed a large piece of bread and has been increasing ever since. The patient has never been hospitalized, has had no surgeries, and is otherwise very healthy. He takes herbal medicines for his prostate on the advice of his neighbor, but with only moderate success according to him. From time to time he also takes a tablet of aspirin for headaches. However, he had not had a headache for quite some time. Today, he has not yet taken any medication. His last tetanus vaccination was 4 years ago.

The patient comes to you in the examination room. He is somewhat overweight. His blood pressure is 130/80 mmHg and his body temperature is 37.1 °C. The pupils are isochoric with prompt direct and indirect light response. The skin looks unremarkable. Heart sounds, abdominal exam, and neurologic exam are unremarkable. On auscultation, you note that the man has no distinct breath sounds on the left side of his chest. The patient complains of severe pain that is sharp and intensifies with deep breathing or coughing. At times, he says, he is dizzy with the pain. His breathing is rapid, with a respiratory rate of 24 breaths per minute. His oxygen saturation is 88% on room air. GCS is 15 (eyes 4/ verbal 5/ motor 6). The lab values show a normal blood count and CRP, and capillary blood glucose is 5.8 mmol/L. After about 10 minutes, the patient states that he is not feeling well at all now. He starts breathing even faster, you measure the blood pressure again, it is now 87/61 mmHg, pulse is 126/min.

**Clinical Case Vignette 2**

You are on weekend duty at the Inselspital. Two colleagues are unfortunately ill with Corona, which is why you have been on duty for many hours. A 62-year-old woman, Mrs. Müller, is admitted to the emergency room. She says she is having more and more trouble breathing. It also hurts her to breathe on the left side of the chest, at about the level of the 6th rib in the medioclavicular line. The patient suffers from hypertension and has been taking two different antihypertensive medications and aspirin for many years. However, she says that none of this is too bad. She has no known history of lung problems or blood clotting disorders; she had a traffic accident with a fracture of her lower leg years ago and a bowel obstruction 10 years ago. She is allergic to bees and any flowers.

On physical examination, you note that the patient is breathing rapidly, has shortness of breath, and is almost unable to speak due to shortness of breath. The patient complains of respiratory chest pain more on the left than on the right. No unusual heart sounds or breath sounds are heard. The abdomen is mildly distended but without pain on palpation, normal bowel sounds; she last drank a bit of coffee and ate a piece of bread with butter 2 hours ago. The scar from bowel obstruction surgery is about 10 cm long and non-irritant with no evidence of acute infection.

You collected vital signs. GCS is 15, blood pressure is 105/70mmHg and heart rate is 100/min. The temperature measured in the ear is 37.1°C. Body weight is 100 kilograms and height 170 centimeters.

ECG reveals sinus tachycardia with a heart rate of 110 beats per minute and an S1Q3 type, PQ time and excitation regression are unremarkable. Blood sampling reveals an elevated D-dimer concentration on point-of-care testing, a CRP of 17 mg/L, normal renal function, and a normal blood count except for leukocytes of 13 G/L.

Just as you are about to check the blood glucose, the patient suddenly moans, saying that she is now very dizzy. You are able to lay her down on the examination table just in time. Her radial pulse is now very fast and only faintly palpable. You see a clear use of the respiratory support muscles, the patient breathes very fast, approx. over thirty breaths / minute.
