# Supplementary material for: Interprofessional Team Training With Virtual Reality: Acceptance, Learning Outcome, and Feasibility Evaluation Study
Source: JMIR Serious Games. 2024 Nov 4;12:e57117. doi: 10.2196/57117 (PMC11554288; doi:10.2196/57117)
Supplement: Multimedia Appendix 3 [file games-v12-e57117-s003.docx]

| **Handover Assessment Tool**  **(Adapted)** |  | **Pre-course** | **Post-course** | **Student ID** |
| --- | --- | --- | --- | --- |
|  |  |  |  |  |
|  | |  |  |  |
| **Introduction** | | **Observed** | **Not observed** | **Comments** |
| Introduces self | |  |  |  |
| Introduces patient | |  |  |  |
| **Situation** | |  |  |  |
| Identifies and communicates the main concern | |  |  |  |
| Clear evidence of priorisation of issues | |  |  |  |
| **Background** | | | | |
| Communicates a well-structured history | |  |  |  |
| Key factors from history are all present | |  |  |  |
| **Assessment** | | | | |
| Communicates appropriate exam findings | |  |  |  |
| Gives details of additional investigative findings | |  |  |  |
| Communicates important aspects of treatment started | |  |  |  |
| **Recommendation** | | | | |
| It is clear from recommendation what the student expects from the recipient - eg - review, advice, transfer | |  |  |  |
| **Overall** | | | | |
| Has the student communicated the handover in a clear and logical manner? | |  |  |  |
| Has the student communicated a concise handover of the patient? | |  |  |  |

Adapted from:

Holt, N., Crowe, K., Lynagh, D., & Hutcheson, Z. (2020). Is there a need for formal undergraduate patient handover training and could an educational workshop effectively provide this? A proof-of-concept study in a Scottish Medical School. *BMJ Open*, *10*(2), e034468. https://doi.org/10.1136/bmjopen-2019-034468
