# Supplementary material for: Interprofessional Team Training With Virtual Reality: Acceptance, Learning Outcome, and Feasibility Evaluation Study
Source: JMIR Serious Games. 2024 Nov 4;12:e57117. doi: 10.2196/57117 (PMC11554288; doi:10.2196/57117)
Supplement: Multimedia Appendix 4 [file games-v12-e57117-s004.docx]

| **Codes** | **Subcodes** | **Examples** |
| --- | --- | --- |
| **Suggestions for improvement** | | |
| **e-Learning** | | |
| Content (6) | Expand (5) | "Too little knowledge coverage in e-learning, too superficial". |
|  | Overload (1) | "A too big topic for the short time of e-learning". |
| Ressources (1) | Share knowledge (1) | "Slides were not provided (with additional links, etc.)" |
| *Positive feedback (4)* | *Content (2)* | *"short and to the point, very good"* |
|  | *Refresher (2)* | *"ISBAR, headache and epilepsy should be known from studies. Refresher before simulation very useful. Focused on the most essential."* |
| **VR Simulation** | | |
| Technical Issues (3) | Differentiating System error / real (2) | "In part, it wasn't entirely clear whether system error occurred or real problem, finding a solution here would be perfect." |
|  | Technical limitation (1) | "Basically well constructed, individual technical limitations" |
| Scenario (3) | Duration (2) | "Too little time to talk to the patient from a doctor's perspective, seizure comes so fast I didn't have time to even perform a neurological exam" |
|  | Content (1) | "Medication or dosage reference book in VR Room." |
| *Self-reflection (2)* |  | *"I think that was more on me than on the e-learning."* |
| *Positive (1)* |  | *"Good repetition of a scheme already known"* |
| **Debriefing** | | |
| Content (2) | Teamwork Skills (1) | "Going through a teamwork questionnaire" |
|  | Detail (1) | "Again, lacking a little detail for me, not enough expertise imparted." |
| Structure (1) |  | "A better structure. E.g. 1. experience and feeling, 2. professional reflection". |
| *Positive (6)* |  | *"Pleasant debriefing, open to comments and feedback."* |

| **Codes** | **Subcodes** | **Examples** |
| --- | --- | --- |
| **Key learning experience** | | |
| Communication (16) | Communication (in emergency) (8) | "Communication represents a very important player in emergency response, it doesn't matter who takes the lead" |
|  | Communication and Teamwork (4) | "Communication is very important. Only when communication is present can good cooperation take place." |
|  | Hands-off communication (3) | "Communication and 10 sec hands off is the most important." |
|  | Feedback (1) | "Feedback communication" |
| Handover (6) | Handover (and stress) (6) | "A good handover is very important. Saves much more time overall than if everything is asked twice." |
| Inter-professional Work / Teamwork (5) |  | "Interprofessional collaboration" |
| Composure (4) |  | "Keeping a cool head when things get stressful." |
| Structure (3) | Structure in thinking (2) | "Structured thinking and action" |
|  | Schemata (1) | "The schemes and processes I already know are good - I can rely on them" |
| Importance of Training (3) | Emergency setting (3) | "Emergency situation simulation and interdisciplinary work". |
| VR as a learning tool (3) | Satisfaction (2) | "I've never had anything to do with VR before and am now generally excited about the possibilities of VR. The biggest learning experience is that VR can enrich learning immensely." |
|  | cognitive load (1) | "Dealing with VR has already taken a lot of cognitive effort, which highlights the importance of schemas and mnemonic devices to be able to react even under stress" |
| Psychological Safety (2) | Ask for help (2) | "I still feel very insecure in emergency situations and with medication. Communication is very important. Just having the confidence to ask when I'm unsure." |

| **Codes** | **Subcodes** | **Examples** |
| --- | --- | --- |
| **Comments** | | |
| Technical (20) | Audio (7) | "The audio had an echo, voices were too quiet, making communication more challenging." |
|  | Controls (5) | "Specific tasks in the simulation were not as intuitive as expected, leading to brief moments of frustration.  (For example, reattaching the oxygen tube when the mask was removed)" |
|  | Blurred vision (4) | "The image was mostly blurry and exhibited significant flickering." |
|  | Issues (4) | “Throughout the simulation, there was a persistent gray area in the field of vision (a technical issue), which was highly distracting and impeded the workflow.” |
| Avatar (3) | Realism (2) | "For team collaboration, more human-like avatars would likely be less awkward." |
|  | Perception (1) | "Because only hands and the head are present, one perceives the other person much less." |
| Language (3) | German preferred (3) | "An optimization could include using Swiss German in the simulation, as it would be more true to life. It would also be more practical if the patient could speak in German.” |
| VR = New (6) | Repetition desired (4) | "It would be interesting to know how one performs and beats in a 2nd VR training. This is because I can imagine that the 'novelty' of this simulation required a significant amount of concentration and attention, potentially affecting my ability to focus on the emergency simulation immediately." |
|  | Stress/overload (2) | "Since I had never worked with a VR headset before, it was somewhat challenging. The 'unfamiliar' environment made me a bit nervous and stressed, which may have slightly affected my performance during the simulation." |
| Scenario (13) | Orientation/instruction needed (6) | "Please test every function during the introduction to ensure that no questions arise afterward." |
|  | Unfamiliar teammates (2) | "It also made it more challenging that the nurse and I didn't know each other..." |
|  | Duration (2) | "The scenario felt a bit too brief for me, so it felt like I didn’t fully immerse myself" |
|  | No replacement of real simulator (1) | "More simulation bias than a 'real' simulator. It would be great if this format of simulation could be used more frequently! From my perspective, it doesn't replace the real simulator." |
| VR as a learning tool (20) | Overall (11) | "The simulation was extremely helpful in recognizing personal limitations and identifying knowledge gaps. It allows for the practice of theoretical knowledge in a practical manner." |
|  | For team training (4) | "For team training, it is very beneficial, but the hands-on skills are not learned through this." |
|  | Realistic (3) | "The idea of calling the attending physician came late; I believe in reality, I would have been more aware of their presence and would have done so at the onset. In this regard, the situation didn't feel entirely realistic. Nevertheless, everything feasible was executed quite well, creating an almost 'real' experience." |
|  | Standards/schemata (2) | "I believe that this tool is not helpful for learning communication, but it's excellent for learning procedures and standards." |
